# What Do You Do It For?

**Published:** 1880-07

**Authors:** H. E. Beach


### WHAT DO YOU DO IT FOR?

*Read before the Tenn. State Dental Society.*

BY H. E. BEACH.

It might be inferred from the caption of this article that direct reference is had to some particular individual, whose views happen to come in conflict with the eclectic element of the mind of its writer, but such is not the case.

In these days the progress in dentistry is so rapid, that it is with great difficulty that men are able to discriminate between the good and the bad, the really valuable and the worthless things that are being offered to the profession, as invaluable adjuncts to the first-class dentist. And yet we are more fully convinced every year that none of these things will take the place of that much needed element for preserving teeth, that is, good sound practical judgment, a judgment that will be firm and unyielding in adapting the best means known to accomplish the end in view. If you question the head lights of the dental profession as to the best method for preserving teeth, you will find them as diversified and as radical in their views as it is possible for men to be; and yet they all seem to be conscientious, and candidly affirm that if you will do their way, success must crown your efforts. What do these wise men say? Dr. A. says God in his wisdom knew best and therefore he made teeth, yea, human teeth, exactly as they ought to be; for this reason when they decay they must be restored to their original shape, or, as Dr. A. says, to their "original contour;" and not only must they be restored, but it must be done with Tom, Dick, or Harry's No. 4 gold. Nothing else will do, and if you are going to do the thing up right, you must begin right, that is the rubber dam must first be applied. It matters not where or what sort of cavity you have to deal with; oh, no, stick to the rule and begin right, apply the rubber dam. Then cut down the frail walls with a particular kind of chisel, next with a sharp excavator remove all decayed matter; then with an engine, and sometimes a particular make of engine, shape the cavity properly; oh yes, properly means a good deal. If you want to know the full meaning of this word *properly* as interpreted by Dr. A., just follow him in his discription of his method of doing it in every conceivable nook or corner that a tooth can possibly decay, and when you have learned all he knows about it, then you will understand what he means by properly. This done cut your retaining pits at the right place and exactly the proper depth; then, having your No. 4 gold prepared, and this must be done by rule too, begin by annealing each peace of gold as it is used, and with an electric mallet, (nobody but an old fogy would think of anything but electricity in this fast age) pack your retaining pits until they are well filled, and then build across and join them together, packing all the time in the direction of the walls of the cavity, and on a line with the axis of the root, until every part is well filled, and if the tooth is much broken down, restore it to its natural contour; then finish with anything you like so it is well done. The main work is done, but it must be well finished, remember that. Now gentlemen, if you will do just as I have described you will never have a failure.

But Dr. B. says Dr. A. is an enthusiast, and is talking just to hear himself talk; that half of his, B's, practice is in filling teeth over that A. made jewelry on. Dr. B. knows better than to follow A.'s rule. He fills with gold alone but with non-cohesive, or, as he prefers to call it "soft gold." He knows a thing or two about preparing the cavity too. He separates well with a double V shaped separation, and never ruins his cavities with retaining pits or grooves, nor does he annoy his patients with rubber dam; but his cavities are shaped properly. Here comes that word properly again, but it has a different meaning this time. Dr. B. makes it mean to remove all the decayed matter from the cavity and smooth up the walls with an engine, with the cavity slightly larger at the base, or somewhere in the interior than at the orifice. This being done, begin the filling with a loosely rolled cylinder of Abbey's old-fashioned soft foil, No. 4 or 6, and continue to introduce these soft cylinders until the cavity is full; then with his, B's, peculiar shaped cylinder plugger, begin by forcing it in this soft filling and inserting harder cylinders. Continue this as long as a place can be found into which the smallest of B.'s cylinder pluggers can be forced, and in the last opening force a very hard cylinder, or, as this is the keystone to the whole structure, pack it full of the same sort of gold folded in very narrow strips, and condense well, but do not think the work complete, for the finishing is of great importance. Now to do this begin with the burnisher and burnish hard, every now and then filing or grinding off the surplus until the whole surface of the filling is perfectly even with the cavity walls; then burnish. Rub with corundum strips, lava strips, buckhorn tape; burnish again, and finish with jewler's rouge on chamois skin. This is Dr. B.'s ideal of a perfect filling; one that will preserve a tooth from carious enroachments during a natural lifetime.

Dr. C. is an old man and has seen a great deal. He is a good dentist too, because he says so, and he knows. He has often described the method of making a soft foil filling out of Abby's old-fashioned foil, and described it well too. Then when cohesive gold was born into the profession he took fast hold of it with all his soul, and shouted aloud, "excelsior, excelsior!" And with sponge gold he could make a perfect filling anywhere in the mouth—under saliva a foot deep if necessary—and there was no mistake about it. Alas for Dr. C.! He is so old, non-cohesive gold is so old, and cohesive gold is so old, that he must have something new; so without due reference to the age of his now pet filling material, he seizes on to plastic fillings, and squalls like a mule's father, "new departure, new departure," and all at once makes the discovery that amalgam is the thing. Oxy-chloride is the thing, and gutta percha is the thing; or in plain terms, that plastic filling is the only safe road to success, and then they are so easy to make. All this seems very nice, but Dr. C. looks through his own glasses, and sees everybody's faults but his own.

Dr. D.'s time comes next, he has but little to say outside of chemical science. He sees a galvanic battery in every mouth that has two metals in it; and with his high power lens can see it when there is only one. Therefore he concludes that no metal, not even lead, has any place in the mouth.

Now, gentleman dentist, "What do you do it for?" I am persuaded that some men do it for controversy's sake; others to foster some pet scheme of their own, while others are actuated alone by a desire to be on the popular side, for sordid motives. This seems a little strong, but why condemn others because they do not think and act as you do? Every good dentist is conscientious, and will always use the best means at his command to accomplish his ends. If a tooth must be saved he never thinks to stop and inquire what Dr. A., B., C. or D. says, because they are unsafe guides; but he calmly and deliberately takes in the whole situation, calls up all his experience, and that of others who have sought nothing but the best interests of their patrons, and upon this he makes up his mind what is best. He uses cohesive gold when he thinks it best; non-cohesive gold when his judgment directs it. Amalgam comes in for its place, and tin finds favor with him too. Oxy-chloride and gutta percha are necessary adjuncts. All these are made subservient to his will. I would as soon undertake to practice dentistry with but one filling material as I would physic with one medicine; one seems about as sensible as the other. Then why so much ado about either one of these things? Let each one occupy what it is certainly entitled to—its proper place in every mouth.

Now, gentlemen, inasmuch as two dental colleges are about to be started in our midst, one under the auspices of the University of Tennessee, the other under the Vanderbilt University, let us hope that no one-sided schism will find its way into either of them, for these schisms only tend to distract the minds of the students, and leave them, as it were, as a ship at sea without chart or compass: they have a rudder to steer their bark—that is the ability—but they will not know what course to pursue.

A cultivated mind, clear head, close application to details, careful manipulation, close observation, gentlemanly deportment, constant attention to business, guided by a consicientious discharge of every known duty, will make a good dentist; and add to this firmness tempered with kindness and patience, the same man will be successful.

Teach men to inscribe on their banner, *Spectemur agendo, et non dicendo.*

